# A new look into uveitis in Colombia: changes in distribution patterns and clinical characteristics over the last 25 years

**DOI:** 10.1007/s00417-022-05796-2

**Published:** 2022-08-22

**Authors:** Diego Polanía, Juliana Reyes-Guanes, William Rojas-Carabali, Daniella Pardo-Pizza, Doménico Barraquer-Lopez, Carlos Cifuentes-González, Natalia Neira-Segura, Alejandra de-la-Torre

**Affiliations:** 1 Neuroscience Research Group (NEUROS), Escuela de Medicina Y Ciencias de La Salud, Universidad del Rosario, Bogotá, Colombia; 2 Ophthalmology Interest Group, Escuela de Medicina Y Ciencias de La Salud, Universidad del Rosario, Bogotá, Colombia

**Keywords:** Etiology, Referral pattern, Classification, Uveitis, Colombia

## Abstract

**Purpose:**

To describe the distribution patterns and clinical characteristics of patients diagnosed with uveitis at a specialized uveitis center in Bogotá, Colombia, from 2013 to 2021 and compare these patterns with the previously reported between 1996 and 2006.

**Methods:**

We performed an observational descriptive cross-sectional study systematically reviewing clinical records of patients attending between 2013 and 2021. Data were analyzed and compared with previous reports.

**Results:**

Of the 489 patients with uveitis, 310 were females (63.4%). The mean age of onset was 38.7, with a range between 1 and 83 years. Bilateral (52.8%), anterior (45.8%), non-granulomatous (90.8%), and recurrent (47.6%) were the most common types of uveitis found in our population sample. The most common cause of uveitis in this study was idiopathic, followed by toxoplasmosis and HLA-B27 + associated uveitis, which differs from the previous Colombian study where ocular toxoplasmosis was the most frequent cause. This highlights a significant shift from infectious etiologies to more immune-mediated processes as the cause of uveitis in Colombia nowadays.

**Conclusion:**

The results of this study provide a comparison between the clinical patterns of presentation of uveitis from 1996 to 2006 and the patterns observed from 2013 to 2021, enhancing awareness about the changing dynamics of uveitis in Colombia to guide a better understanding of the diagnosis, classification, and correlation with other systemic conditions of the disease.


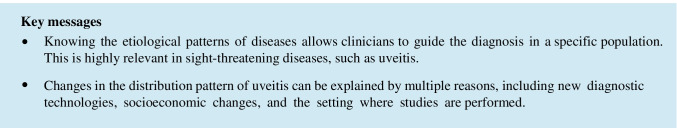


## Background

Uveitis is the inflammation of the uveal tract, which encompasses the ciliary body, choroid, and iris. Nevertheless, it can affect adjacent tissues such as the retina, optic nerve, and vitreous. Uveitis can be related to a local or systemic affection, and its etiologies can be divided between infectious and noninfectious [1, 2].

In 2005, Jabs et al. [3] proposed the Standardization of Uveitis Nomenclature (SUN) to describe the disease better. It includes anatomical classification, uveitis descriptors, and grading schemes for anterior chamber cells, anterior chamber flare, and vitreous haze. This became a beneficial aid for appropriate diagnosis and treatment and helped uvea specialists and ophthalmologists comprehend in better way the patterns and clinical characteristics of uveitis.

Studies in developed countries have reported a uveitis incidence between 17 and 52.4 per 100,000 persons per year and a prevalence of 36.2 to 730 per 100,000 persons [4–7]. Additionally, uveitis causes about 10% of all cases of visual loss in the western world and 5–20% of legal blindness in developed countries [8, 9]. However, in developing countries, it has been reported to cause blindness in up to 25% of the cases [8, 10]. Although uveitis might be present in any age group and sex, several studies have described an age preference from 20 to 50 years and no sex predominance [10–13].

Many studies have shown differences in the clinical manifestation of uveitis in correlation with sex, age, race, genes, socioeconomic factors, environmental exposure, geographical region, and immunological response [11–14]. These studies have been crucial to the early diagnosis and management of the disease [10, 15, 16]. Nevertheless, few epidemiological studies have been performed in South America and only one in Colombia [17]. Therefore, this study aimed to describe the distribution patterns and clinical characteristics of patients with uveitis diagnosis from a specialized uveitis center in Bogotá, Colombia, from 2013 to 2021 and compare these patterns with the previously reported patterns between 1996 and 2006.

## Methods

### Design

We conducted an observational descriptive cross-sectional study based on the STROBE guidelines. The Universidad del Rosario Ethics Committee approved this study.

### Population

The data were obtained from the clinical records of patients evaluated in an ophthalmological referral center specialized in uveitis in Bogotá from March 28, 2013, to February 27, 2021.

### Data collection and statistical analysis

Patient information was gathered from clinical records in a previously validated Excel form, including age, sex, age at onset, age at presentation, clinical diagnosis according to the SUN classification system [3], laterality, course of the disease, grade of inflammation, best-corrected visual acuity (BCVA), type of uveitis, etiologic diagnosis, and complications. The International Classification of Diseases, Tenth Revision (ICD-10) was used to depurate patients’ clinical records in the software. A final diagnostic list was constructed following classic criteria for ocular inflammatory diseases from two reference uveitis textbooks [18, 19]. A single uveitis specialist evaluated all the patients and retrieved the medical records to guarantee the data quality.

All patients underwent a complete ophthalmologic examination that consisted of slit-lamp biomicroscopy, tonometry, indirect ophthalmoscopy, and evaluation of the BCVA. In the same way, all patients were requested for the following tests: complete blood count with erythrocyte sedimentation rate, C-reactive protein, urine analysis, venereal disease research laboratory (VDRL), fluorescent treponemal antibody-absorption (FTA-ABS), purified protein derivate (PPD-Mantoux), or interferon-gamma release assays (IGRAs) and chest radiography. Additional ophthalmic tests (e.g., fluorescein angiography, optical coherence tomography, and visual field testing) were performed when indicated. Other ancillary examinations were carried out when necessary to make diagnoses, including computed tomography, magnetic resonance imaging, HLA-B27/B51/DR4/A29 typing, serum angiotensin-converting enzyme, lysozyme, serum calcium, antinuclear antibodies, antineutrophil cytoplasmic antibodies, extractable nuclear antibodies, rheumatoid factor, anticardiolipin antibodies, fluorescent treponemal antibody absorption test, purified protein derivative, *Toxoplasma* antibodies, *Toxocara* antibodies, *Herpes simplex*, *Herpes zoster*, and *Cytomegalovirus* antibodies*, Borrelia* antibodies, and enzyme-linked immunosorbent assay for HIV. Ocular toxoplasmosis was diagnosed based on clinical criteria as the presence of an active creamy-white focal retinal lesion eventually combined with hyperpigmented retinochoroidal scars in either eye plus positive anti-*Toxoplasma* IgG and/or IgM. Intraocular fluids PCR was requested to confirm atypical cases [20–22].

The Excel database was filled by co-investigators trained in data entry and management to guarantee interobserver unification. The univariate statistical analysis was performed in SPSS using absolute and relative frequencies for categorical variables and mean and standard deviations for continuous variables. In cases where an etiology could not be discovered due to lack of follow-up of the patients, without having ruled out all possible diagnoses, it was considered undetermined. Idiopathic etiology was reserved for cases where the diagnosis could not be determined after ruling out infectious and noninfectious causes of uveitis.

## Results

We reviewed 489 clinical records of patients with uveitis, of which 310 (63.4%) were female. The mean age of onset was 38.7 years, ranging between 1 and 83 years. Bilateral compromise was observed in 52.8% (*n* = 258) and unilateral in 47.2% (*n* = 231). Demographic information of the study population is summarized in (Table 1).

**Table 1:** Demographics of the uveitis study population

Demographics	Anterior uveitis	Intermediate uveitis	Posterior uveitis	Panuveitis	Total
Age (years) mean $\pm$ SD					
At consultation	49 $\pm$ 18	$27 \pm$ 21	$34 \pm$ 21	$41 \pm$ 21	$42.47 \pm$ 21.026
At onset	$45.7 \pm 18.9$	$24.3 \pm$ 21.3	$28 \pm$ 21	$37.5 \pm$ 21.1	$38.7 \pm$ 21.4
Gender *n* (%)					
Female	157	20	47	86	310 (63.4)
Male	67	18	33	61	179 (36.6)
Total, *n* (%)	224	38	80	147	489 (100.0)

Anterior uveitis was the most common localization (*n* = 224, 45.8%), followed by panuveitis (*n* = 147, 30.1%), posterior uveitis (*n* = 80, 16.3%), and intermediate uveitis (*n* = 38, 7.8%). Most cases presented a recurrent course (*n* = 233, 47.6%), insidious onset (*n* = 272, 55.6%), and persistent duration (*n* = 315, 64.4%). Non-granulomatous uveitis was significantly more frequent than granulomatous uveitis (90.8% vs. 9.2%, respectively). Most anterior and posterior uveitis cases were unilateral, in contrast to intermediate and panuveitis, which presented with bilateral compromise in 73% (*n* = 28) and 63.9% (*n* = 94) of cases. More detailed information can be found in Table 2.

**Table 2:** Distribution of uveitis according to different classification criteria

Characteristics	Anterior uveitis	Intermediate uveitis	Posterior uveitis	Panuveitis	Total
	*N* = 224 (%)	*N* = 38(%)	*N* = 80(%)	*N* = 147(%)	*N* = 489 (%)
Ocular involvement					
Bilateral	100 (44.6)	28 (73.7)	36 (45)	94 (63.9)	258 (52.8)
Unilateral	124 (55.4)	10 (26.3)	44 (55)	53 (36.1)	231 (47.2)
Onset					
Insidious	116 (51.8)	28 (73.7)	37 (46.3)	91 (61.9)	272 (55.6)
Sudden	108 (48.2)	10 (26.3)	43 (53.8)	56 (38.1)	217 (44.4)
Duration					
Limited	106 (47.3)	10 (26.3)	21 (26.3)	37 (25.2)	174 (35.6)
Persistent	118 (52.7)	28 (73.7)	59 (73.8)	110 (74.8)	315 (64.4)
Course					
Acute	30 (13.4)	3 (7.9)	12 (15)	15 (10.2)	60 (12.3)
Chronic	67 (29.9)	16 (42.1)	40 (50)	73 (49.7)	196 (40.1)
Recurrent	127 (56.7)	19 (50)	28 (35)	59 (40.1)	233 (47.6)
Type of inflammation					
Granulomatous	15 (6.7)	4 (10.5)	3 (3.8)	23 (15.6)	45 (9.2)
Non-granulomatous	209 (93.3)	34 (89.5)	77 (96.3)	124 (84.4)	444 (90.8)

A specific diagnosis was made in 408 (83.4%) cases. In 81 (16.6%) of the patients, the cause could not be determined. Overall, idiopathic was the most common cause with 145 patients (29.7%), followed by toxoplasmosis with 78 cases (16%) and HLAB-27 + associated uveitis with 27 cases (5.5%). Females were more commonly affected by idiopathic and HLA-B27 + associated uveitis than men. On the other hand, in toxoplasmosis, the sex distribution was very similar (*n* = 40, 51.3% in women vs. *n* = 38, 48.7% in men) (Table 3). Regarding idiopathic uveitis, 68.3% (*n* = 99) were bilateral (Table 3), and in the cases of toxoplasmosis, 62.8% (*n* = 49) were bilateral, whereas 37.2% (*n* = 29) were unilateral.

**Table 3:** Causes of uveitis, laterality, and gender distribution

Diagnosis	n	%	Affected eye		Gender distribution	
			Bilateral	Unilateral	Female	Male
			*n* (%)	*n* (%)	*n* (%)	*n* (%)
Idiopathic	145	29.7	99 (68.3)	46 (31.7)	102 (70.3)	43 (29.7)
Undetermined	81	16.6	34 (42.0)	47 (58)	54 (66.7)	27 (33.3)
Toxoplasmosis	78	16	29 (37.2)	49 (62.8)	40 (51.3)	38 (48.7)
HLA-B27 +	27	5.5	11 (40.7)	16 (59.3)	17 (63.0)	10 (37.0)
Juvenile idiopathic arthritis	13	2.7	9 (69.2)	4 (30.8)	11 (84.6)	2 (15.4)
*Herpes zoster* virus	11	2.2	4 (36.4)	7 (63.6)	6 (54.5)	5 (45.5)
*Herpes simplex* virus	11	2.2	0	11 (100)	6 (54.5)	5 (45.5)
Sjögren syndrome	10	2	3 (30)	7 (70)	9 (90.0)	1 (10)
Vogt–Koyanagi–Harada syndrome	9	1.8	8 (88.9)	1 (11.1)	6 (66.7)	3 (33.3)
Ankylosing spondylitis	9	1.8	5 (55.6)	4 (44.4)	6 (66.7)	3 (33.3)
Non-specific viral	7	1.4	2 (28.6)	5 (71.4)	3 (42.9)	4 (57.1)
Tuberculosis	6	1.2	6 (100)	0	4 (66.7)	2 (33.3)
Ulcerative colitis	5	1	4 (80)	1 (20)	3 (60)	2 (40)
Rheumatoid arthritis	5	1	3 (60)	2 (40)	5 (100)	0
Multiple sclerosis	5	1	4 (80)	1 (20)	4 (80)	1 (20)
Sarcoidosis	4	0.8	3 (75)	1 (25)	4 (100)	0
Reactive arthritis	4	0.8	2 (50)	2 (50)	0	4 (100)
Axial spondyloarthritis	4	0.8	3 (75)	1 (25)	2 (50)	2 (50)
TINU syndrome	3	0.6	2 (66.7)	1 (33.3)	2 (66.7)	1 (33.3)
Possner–Schlossman syndrome	3	0.6	1 (33.3)	2 (66.7)	2 (66.7)	1 (33.3)
Peripheral spondiloarthritis	3	0.6	2 (66.7)	1 (33.3)	2 (66.7)	1 (33.3)
Endophthalmitis	3	0.6	1 (33.3)	2 (66.7)	1 (33.3)	2 (66.7)
Drug-induced uveitis	3	0.6	2 (66.7)	1 (33.3)	3 (100)	0
*Cytomegalovirus*	3	0.6	1 (33.3)	2 (66.7)	1 (33.3)	2 (66.7)
Toxocariasis	2	0.4	0	2 (100)	1 (50)	1 (50)
Systemic lupus erythematosus	2	0.4	2 (100)	0	2 (100)	0
Suspected Behçcet	2	0.4	1 (50)	1 (50)	1 (50)	1 (50)
Syphilis	2	0.4	0	2 (100)	2 (100)	0
Serpiginous choroiditis	2	0.4	2 (100)	0	1 (50)	1 (50)
Relapsing polychondritis	2	0.4	1 (50)	1 (50)	2 (100)	0
Psoriatic arthritis	2	0.4	2 (100)	0	0	2 (100)
Psoriasis	2	0.4	1 (50)	1 (50)	0	2 (100)
Probable Sarcoidosis	2	0.4	2 (100)	0	1 (50)	1 (50)
Post-traumatic uveitis	2	0.4	0	2 (100)	0	2 (100)
Sympathetic ophthalmia	2	0.4	0	2 (100)	1 (50)	1 (50)
*Epstein Barr* virus	2	0.4	1 (50)	1 (50)	1 (50)	1 (50)
Birdshot retinochoroidopathy	2	0.4	2 (100)	0	2 (100)	0
Antiphospholipid syndrome	2	0.4	1 (50)	1 (50)	0	2 (100)
*Human immunodeficiency* virus (HIV) retinopathy	1	0.2	1 (100)	0	0	1 (100)
Cryoglobulinemia vasculitis	1	0.2	0	1 (100)	1 (100)	0
Suspected leptospirosis	1	0.2	1 (100)	0	0	1 (100)
Postoperative uveitis	1	0.2	0	1 (100)	1 (100)	0
Masquerade syndrome	1	0.2	0	1 (100)	0	1 (100)
Mixed connective tissue disease	1	0.2	1 (100)	0	0	1 (100)
Crohn’s disease	1	0.2	1 (100)	0	1 (100)	0
Suspected Brucellosis	1	0.2	0	1 (100)	0	1 (100)
Blau syndrome	1	0.2	1 (100)	0	0	1 (100)
**Total**	489	100	258 (52.8)	231 (47.2)	310 (63.4)	179 (36.6)

In patients with anterior and intermediate uveitis, idiopathic uveitis was the most common etiology (*n* = 67, 29.9% and *n* = 21, 55.3%, respectively) (Table 4). HLA-B27 + associated uveitis was the second most frequent cause in both groups. Lastly, toxoplasmosis was the most common cause of posterior uveitis and panuveitis (*n* = 57, 71.3%, and *n* = 51, 34.7%, respectively) (Table 4).

**Table 4:** Causes of anterior, intermediate, posterior, and panuveitis

Diagnosis	Anterior	Intermediate	Posterior	Panuveitis
	*N* = 224 (%)	*N* = 38 (%)	*N* = 80 (%)	*N* = 147 (%)
Idiopathic	67 (29.9)	21 (55.3)	6 (7.5)	51 (34.7)
Undetermined	44 (19.6)	5 (13.2)	8 (10)	24 (16.3)
Toxoplasmosis	0	1 (2.6)	57 (71.3)	20 (13.6)
HLA-B27 +	21 (9.4)	3 (7.9)	0	3 (2)
Juvenile idiopathic arthritis	7 (3.1)	2 (5.3)	0	4 (2.7)
*Herpes zoster* virus	8 (3.6)	0	0	3 (2)
*Herpes simplex* virus	10 (4.5)	0	0	1 (0.7)
Sjögren syndrome	7 (3.1)	0	0	3 (2)
Vogt–Koyanagi–Harada syndrome	2 (0.9)	0	0	7 (4.8)
Ankylosing spondylitis	8 (3.6)	0	0	1 (0.7)
Non-specific viral	4 (1.8)	0	0	3 (2)
Tuberculosis	0	1 (2.6)	2 (2.5)	3 (2)
Ulcerative colitis	5 (2.2)	0	0	0
Rheumatoid arthritis	4 (1.8)	0	0	1 (0.7)
Multiple sclerosis	2 (0.9)	1 (2.6)	1 (1.3)	1 (0.7)
Sarcoidosis	0	1 (2.6)	0	3 (2)
Reactive arthritis	2 (0.9)	0	0	2 (1.4)
Axial spondylarthritis	4 (1.8)	0	0	0
TINU syndrome	1 (0.4)	0	0	2 (1.4)
Possner–Schlossman syndrome	3 (1.3)	0	0	0
Peripheral spondylarthritis	3 (1.3)	0	0	0
Endophthalmitis	2 (0.9)	0	0	1 (0.7)
Drug-induced uveitis	3 (1.3)	0	0	0
Cytomegalovirus	2 (0.9)	0	1 (1.3)	0
Toxocariasis	0	0	0	2 (1.4)
Systemic lupus erythematosus	2 (0.9)	0	0	0
Suspected Behçet	1 (0.4)	0	0	1 (0.7)
Syphilis	1 (0.4)	0	1 (1.3)	0
Serpiginous choroiditis	0	0	2 (2.5)	0
Relapsing polychondritis	2 (0.9)	0	0	0
Psoriatic arthritis	1 (0.4)	0	0	1 (0.7)
Psoriasis	0	0	1 (1.3)	1 (0.7)
Probable sarcoidosis	0	2 (5.3)	0	0
Post-traumatic uveitis	1 (0.4)	0	0	1 (0.7)
Sympathetic ophthalmia	0	0	1 (1.3)	1 (0.7)
*Epstein Barr* virus	0	0	0	2 (1.4)
Birdshot retinochoroidopathy	0	0	0	2 (1.4)
Antiphospholipid syndrome	1 (0.4)	0	0	1 (0.7)
*Human immunodeficiency* virus (HIV) retinopathy	0	0	0	1 (0.7)
Cryoglobulinemic vasculitis	1 (0.4)	0	0	0
Suspected leptospirosis	0	1 (2.6)	0	0
Postoperative uveitis	1 (0.4)	0	0	0
Masquerade syndrome	1 (0.4)	0	0	0
Mixed connective tissue disease	1 (0.4)	0	0	0
Crohn’s disease	1 (0.4)	0	0	0
Suspected Brucellosis	1 (0.4)	0	0	0
Blau syndrome	0	0	0	1 (0.7)
**Total**	224 (100)	38 (100)	80 (100)	147 (100)

Finally, panuveitis was the most common uveitis in patients under 16 years. Anterior uveitis was the most common in patients between 16 and 50 years and in the group over the age of 50 years. Idiopathic uveitis was the most common diagnosis in all age groups, followed by toxoplasmosis (Tables 5 and 6).

**Table 5:** Distribution of anatomical forms of uveitis according to age

Age	Anterior	Intermediate	Posterior	Panuveitis	Total*
	*N* = 214 (%)	*N* = 37 (%)	*N* = 75 (%)	*N* = 135 (%)	
Under 16 years	12 (16.2)	18 (24.3)	19 (25.7)	25 (33.8)	74
16–50 years	89 (44.7)	12 (6)	39 (19.6)	59 (29.6)	199
Over 50 years	113 (60.1)	7 (3.7)	17 (9)	51 (27.1)	188

*28 patients had not reported the age in the clinical record

**Table 6:** Distribution causes of uveitis by age

Diagnosis	< 16	16–50	> 50
	*N* = 74 (%)	*N* = 199 (%)	*N* = 188 (%)
Ankylosing spondylitis	0	4 (2)	5 (2.7)
Antiphospholipid syndrome	0	2 (1)	0
Axial spondylarthritis	0	0	4 (2.1)
Birdshot retinochoroidopathy	0	0	2 (1.1)
Blau syndrome	1 (1.4)	0	0
Suspected Brucellosis	0	1 (0.5)	0
Crohn’s disease	0	0	1 (0.5)
*Cytomegalovirus*	0	2 (1)	1 (0.5)
Drug-induced uveitis	0	0	3 (1.6)
Endophthalmitis	0	0	3 (1.6)
Mixed connective tissue disease	0	0	1 (0.5)
*Epstein Barr* virus	0	1 (0.5)	1 (0.5)
*Herpes simplex* virus	1 (1.4)	2 (1)	6 (3.2)
*Herpes zoster* virus	1 (1.4)	5 (2.5)	5 (2.7)
HLA-B27 +	3 (4.1)	13 (6.5)	9 (4.8)
Idiopathic	30 (40.5)	55 (27.6)	52 (27.7)
Juvenile idiopathic arthritis	10 (13.5)	3 (1.5)	0
Multiple sclerosis	0	5 (2.5)	0
Sympathetic ophthalmia	0	1 (0.5)	1 (0.5)
Peripheral spondyloarthritis	0	2 (1)	1 (0.5)
Possner–Schlossman syndrome	0	2 (1)	1 (0.5)
Postoperative uveitis	0	0	1 (0.5)
Post-traumatic uveitis	0	2 (1)	0
Probable sarcoidosis	1 (1.4)	0	1 (0.5)
Psoriasis	0	1 (0.5)	1 (0.5)
Psoriatic arthritis	0	0	2 (1.1)
Reactive arthritis	0	4 (2)	0
Relapsing polychondritis	0	1 (0.5)	1 (0.5)
Rheumatoid arthritis	0	3 (1.5)	2 (1.1)
Sarcoidosis	0	1 (0.5)	3 (1.6)
Serpiginous choroiditis	0	1 (0.5)	1 (0.5)
Syphilis	0	1 (0.5)	1 (0.5)
Sjögren syndrome	0	1 (0.5)	8 (4.3)
Suspected Behçet	0	1 (0.5)	1 (0.5)
Suspected leptospirosis	1 (1.4)	0	0
Systemic lupus erythematosus	0	0	2 (1.1)
TINU syndrome	1 (1.4)	1 (0.5)	1 (0.5)
Toxocariasis	1 (1.4)	1 (0.5)	0
Toxoplasmosis	14 (18.9)	37 (18.6)	21 (11.2)
Tuberculosis	2 (2.7)	3 (1.5)	1 (0.5)
Ulcerative colitis	0	2 (1)	3 (1.6)
Undetermined	8 (10.8)	30 (15.1)	37 (19.7)
Cryoglobulinemia vasculitis	0	1 (0.5)	0
*Human immunodeficiency* virus (HIV) retinopathy	0	1 (0.5)	0
Non-specific viral	0	2 (1)	4 (2.1)
Vogt–Koyanagi–Harada syndrome	0	7 (3.5)	1 (0.5)

## Discussion

This is the second retrospective study done in Colombia with a large population of uveitis patients seen in an ophthalmology referral center. The mean age of uveitis onset was 38.7 years, similar to studies performed in Chile and Brazil [23, 24]. However, the mean age was slightly higher than in our previous Colombian study, where the average age of onset was 31.7 years. This could be explained due to an increase in life expectancy. According to the DANE (National Administrative Department of Statistics), the entity responsible for producing official statistics in Colombia, 13.4% of the country’s total population currently corresponds to people aged 60 years or older. That shows that the elderly population has been steadily increasing over the years, as in 1995, it reached just 7% [25].

Regarding sex distribution, Miserocchi et al. [9] described no predominance, with both sexes being equally affected by uveitis in most of the series. However, when we analyzed the distribution based on the World Bank classification, in the countries classified as high-income economies, such as Japan, England, and Germany, the predominance of cases occurs in females [26–29]. Also, this pattern tends to occur in upper-middle-income countries such as Brazil [24], Thailand [30], and currently in Colombia. However, in countries classified as low-middle income, the predominance is towards the male sex, as occurs in India, Tunisia, and in our previous study in Colombia, when it was classified in this group [17, 31, 32]. This phenomenon is interesting to explore in future studies, possibly explained by more cases due to infectious etiologies or disparities in the access to health between sexes [33].

Additionally, we found that the most common type of uveitis was bilateral, anterior, recurrent, and non-granulomatous, similar to most studies around the world [9]. However, these differ from our previous results, where unilateral, posterior, and acute uveitis were the most common clinical characteristics, which could be explained because toxoplasmosis was the most frequent etiology at that time [34].

According to previous studies of ocular toxoplasmosis (OT) in our country, this disease has been increasing. Indeed, Gomez-Marin et al. described an OT prevalence of 10.5% in 2021 [35], which indicates an increase of 4.5 points compared to another study reporting a prevalence of 6% in 2007 [36]. In the same way, Cifuentes-González et al. found an increasing trend in toxoplasmosis incidence over the last 5 years [37]. The lesser number of OT cases evidenced in the current study could be explained because the clinical records included were retrieved only in one ophthalmic center of the two included in 2009 [17], which corresponds to a private clinic in Bogotá that predominantly attends patients of high socioeconomic status and the socioeconomic conditions have been described as determinants in the prevalence of systemic and ocular toxoplasmosis [38, 39]. Additionally, the other ophthalmic center, of which we did not have access to the medical records on this occasion, is specialized in the retina. This could also explain the lesser number of cases of toxoplasmosis and toxocariasis.

The most common etiology of uveitis in the current study was idiopathic, similar to studies performed primarily in developed countries, where idiopathic uveitis corresponded to 30–60% of the cases [6, 7, 40, 41]. It was followed by toxoplasmosis in second place and HLA-B27 + associated uveitis as the next most frequent cause. This could be since we have more diagnostic resources at our disposal nowadays, making the identification of HLA-B27 + cases possible and accessible to the general public.

We found idiopathic uveitis as the most common cause of anterior uveitis, similar to our previous findings and studies worldwide [42]. However, our current study found HLA-B27 + as the second most frequent etiology of anterior uveitis, which differs from our 2009 study where it was herpes simplex. Nevertheless, it must be considered that HLA-B27 typing was not easily accessible at that time in Colombia, and thus it was not routinely measured in uveitis patients. Our results are consistent with the literature in Asia, where many studies report idiopathic as the most common cause of anterior uveitis [12, 30, 40, 43–46]. Nonetheless, two of them report HLA-B27 as the most common cause [47, 48].

Regarding the etiology of posterior uveitis and panuveitis, toxoplasmosis has remained stable as the primary cause through time, compared to our previous study. Thus, considering the importance of OT as a cause of uveitis in Colombia [17, 34], we used clinical and serological criteria and intraocular fluids PCR in atypical cases to prevent a diagnostic bias. Similarly, toxoplasmosis was found to be the most common cause of posterior uveitis in Argentina [49], India [31], Philippines [45], France [8], Germany [29], Tunisia [32], and Australia [7].

Meanwhile, intermediate uveitis was most frequently associated with an idiopathic cause in both of our studies, followed by HLA-B27 + associated uveitis. Likewise, studies in Argentina [49], Turkey [12], Thailand [30], India [43], Sri Lanka [44], Philippines [45], Taiwan [47], Vietnam [46], Italy [1], France [8], Germany [29], and Tunisia [32] have found idiopathic to be the most common cause. In contrast, a study performed in Chile showed sarcoidosis as the second most frequent cause of intermediate uveitis [23]. The prevalence of sarcoidosis in Colombia has not been determined yet; however, it seems to be infrequent since there are just a few reports and case series in the literature, and we only found 4 cases of confirmed sarcoidosis and 2 suspected cases [50, 51]. This low prevalence could be explained because of the high genetic heterogeneity of the present-day populations in Colombia, considering that multiple alleles of susceptibility and epigenetic factors have been described in cohorts with European- and African-American ancestry [52–54].

Regarding age, in the group under 16 years, posterior uveitis was the most common anatomic localization of the disease in our previous study, but the tendency changed to panuveitis in the current results, which could be explained by the increase in idiopathic cases.

Anterior uveitis was the most common diagnosis in patients aged between 16 and 50 years, similar to results obtained by Liberman et al. [23], who showed a bimodal distribution, with one peak being around age 18 and a second one around age 55. However, it should be noted that in our previous study, panuveitis was the most frequent site of inflammation in this age group. In patients over 50 years, anterior uveitis remained the predominant anatomic type of uveitis, which could be related to the maintenance of idiopathic cases as the most common cause, followed by HLA-B27 + associate uveitis.

The group in which we found the greatest number of patients with uveitis was between 16 and 50 years, which corresponds to a large percentage of the economically active population in Colombia. Therefore, uveitis could have a tremendous socioeconomic impact in our country [55].

In this study, a specific diagnosis was achieved in 83.4% of the cases, with only 16.6% remaining undetermined. This shows a relevant improvement since our previous study, where 21.1% of the patients remained with an unknown cause after the initial examinations [17]. These findings are consistent with the greater availability of confirmatory laboratory studies nowadays in Bogotá, along with more specialized clinicians with a better understanding of the pathophysiological processes of uveitis and how to diagnose and classify the disease.

In Table 7, we present a comparison of clinical features between different published studies around the world.

**Table 7:** Different causes of various types of uveitis reported from different parts of the world

Country (years) [year of publication]	*n*	AU (%) Principal causes	IU (%) Principal causes	PU (%) Principal causes	PAN (%) Principal causes	Infectious (%) Principal causes	Noninfectious (%) Principal causes	Idiopathic (%)
South America								
Brazil [10] (1987–2007) [2013]	862	0.3%	0.1%	91.9%	3.1%	91.8% OT 88.7% CMV 1.0% Congenital rubeolla 1.0%	4.3% Behçet 1.4% VKH 1.3% SO 0.3%	3.9%
Colombia [17] (1996–2006) [2009]	693	28.9% Idiopathic 25.5% HSV 5.5% Lens induced 4.5%	4.3% Idiopathic 96.7% Sarcoidosis 3.3%	35.9 OT 67.2% Toxocariasis 14.6% Idiopathic 3.6%	30.9% OT 46.6% Idiopathic 31.5% VKH 3.6%	-	-	-
Chile [23] (2002–2012) [2014]	611	40.4% Idiopathic 46.6% HLA-B27 10.5% HSV 8.9% DM-associated 6.9%	8.3% Idiopathic 94.1% Sarcoidosis 5.9%	18.0% Idiopathic 43.6% OT 21.8% Toxocariasis 8.2%	33.2% VKH 51.7% OT 7.4% SO 6.4%	28.7% OT 6.4% HSV 3.9% TB 2.3%	71.3% VKH 17.2% HLA-B27 4.3% DM-associated 2.8%	41%
Brazil [24] (2012–2013) [2015]	1053	29.25%	7.22%	40.08%	15.97%	46.34%6 OT 24.03% Syphilis 6.08% TB 5.22%	NR VKH 7.5% JIA 6.36% HLA-B27 6.36%	Anterior idiopathic 9.88% Diffuse idiopathic 1.8%
Argentina [49] (2011–2015) [2022]	212	50% Idiopathic 30% Herpes 21.4% HLA-B27 18.6%	9% Idiopathic 70% Sarcoidosis 1.8%	32% OT 25% Idiopathic 13.2% Behçet and sarcoidosis 5.6% each	8% VKH 29.4% TB 17.6% Sarcoidosis, Behçet, and HLA-B27 11.7% each	36% Herpes 35% OT 23.3% PSS 9.0%	64% (Including idiopathic) Idiopathic 42.2% HLA-B27 18.5% Sarcoidosis 8.1%	28%
North America								
Northern California [4] (1998–1999) [2004]	382	70.6%	2.87%	2.61%	4.97%	-	-	-
Hawaii [6] (2006–2007) [2013]	224	72%	6%	They combined posterior with panuveitis, representing 21%	-	NR HSV/HZV19% Histoplasmosis7% OT: 4%	NR Rheumatoid arthritis 4% Psoriasis 3% IBD 3%	-
ASIA								
Turkey [12] (1990–2010) [2014]	1028	42% Idiopathic 31.5% Herpes 15.5% FUS 11.6%	8.4% Idiopathic 73.2% Behçet 18.6% Masquerading 6.9%	24.9% Behçet 46.1% OT 27% Idiopathic 10.2%	24.7% Behçet 61.0% Idiopathic 27.6% TB 4.6%	14% OT 7.2% Herpes 6.8%	60.3% Behçet 32.2% FUS 5% HLA-B27 4.6%	Idiopathic 25.7%
Tokyo [27] (2016–2018) [2021]	732	33.1%	1.5%	7.1%	58.3%	NR Herpes 6.7% Bacterial endophthalmitis 2.9% PSS 2.6%	NR Sarcoidosis 8.9% Intraocular lymphoma 5.5% VKH 4.8%	NR
China [40] (2008–2011) [2015]	199	51.8% Idiopathic 33.7% Zoster/simplex Keratouveitis 4.0% Traumatic 4.0%	1.0%	14.6% Idiopathic 11.6% OT 1.0% Eales disease 1.0%	32.7% Behçet 10.1% VKH 9.5% Idiopathic 4.5%	7.5% Zoster simplex Keratouveitis 53.3% Infectious endophthalmitis 20% TB and OT 13.3%, each	41.7%	50.8%
Thailand [30] (2007–2012) [2014]	446	44.8% Idiopathic 73.5% Herpes 13% HLA-B27 4.5%	0.9% Idiopathic 100%	14.3% Idiopathic 35.9% VKH 23.4% Behçet 7.7%	40% VKH 47.8% Idiopathic 23.6% Behçet 12.9%	25.7% of the 51.6% with an established diagnosis HSV/VZV 5.8%	74.3% of the 51.6% with an established diagnosis VKH 22.4%	Idiopathic:48.4%
India [31] (2005–2005) [2009]	308	47.07% Idiopathic 45.51% Seronegative spondyloarthropathy 23.44% Traumatic 17.24%	12.98% Pars planitis 77.5% Sarcoidosis 12.5% TB 10%	29.87% OT 40.21% Idiopathic choroiditis 19.56% Serpiginous Choroiditis 15.21%	10.06% VKH 45.16% Sarcoidosis 29% Idiopathic 16.12%	-	-	38.9%
India [43] (2011–2014) [2016]	1912	43.0% Idiopathic 41.7% HLA-B27 22.0% FUS 17.1%	10.7% Idiopathic 44.6% TB 43.1% Sarcoidosis 9.31%	24.6% TB 48.7% Idiopathic 36.6% CMV 3.4%	16.2% TB 29.0% Idiopathic 23.5% VKH 18.4%	33.4%	66.6%	39.4%
Sri Lanka [44] (2010–2014) [2017]	750	38% Idiopathic 66% SSS 13% HLA-B27 8.8%	20% Idiopathic 78% TB 8.0% Sarcoidosis 6.7%	25% Idiopathic 55.6% TO 18.2% TB 11.2%	17% Idiopathic 60.2% Sarcoidosis 14.1% TB 10.9%	17% TB 35.9% TO 29.8% Herpetic 13.7%	83% Sarcoidosis 33.6% SSS 27.6% HLA-B27 18.7%	64.7%
Philippines [45] (2010–2015) [2017]	595	49.6% Idiopathic 75.6% FUS 6.1% TB and HSV 3.73%	6.9% Idiopathic 85.4% TB 14.6%	20.2% TO 37.5% Idiopathic 25.8% TB 19.2%	23.3% VKH 36.7% Idiopathic 24.5% TB 21.6%	25.6% TB 46.1% TO 29.6% Toxocariasis 7.9%	74.4% VKH 12.4% FUS 4.1% SO 2.9%	54.3%
Taiwan [47] (2001–2014) [2016]	450	61.3% HLA-B27 38.8% Idiopathic 25.4% SSS 13.4%	5.8% Idiopathic 92.3% Sarcoidosis 3.8% Lymphoma 3.8%	7.8% VKH 45.7% Idiopathic 11.4% MEWDS 11.4%	25.1% VKH 23.9% Idiopathic 18.6% Behçet 12.4%	16.2% Endophthalmitis 19.2% HSV 17.8% RNA 12.3%	83.7% HLA-B27 29.7% VKH 12.4% PSS 6.2%	26.4%
Vietnam [46] (2011–2015) [2017]	212	46% Idiopathic 55.1% Herpetic 12.2% PSS 11.2%	14.2% Idiopathic 66.7% Sarcoidosis 13.3% TB 10%	22.2% Toxocariasis 25.5% VKH 21.3% TB 17%	17.5 VKH 54.1% Behçet 16.2% TB 13.5%	27.4% TB 32.7% Herpetic 20.7% Toxocariasis 20.7%	72.6% VKH 19.5% Behçet 9.1% PSS 7.1%	36.3%
Singapore [48] (1997–2010) [2017]	1249	64.3% HLA-B27 29% Idiopathic 24% CMV 12.8%	7.4% Idiopathic 73.9% TB 10.9% Lymphoma 7.6%	10.2% CMV 20.5% Idiopathic 17.3% TO and Dengue 16.5%	18.1% VKH 48.2% Idiopathic 12.85 TB 10.2%	31% CMV 33.5% HSV 21.8% TB 21.8%	69% HLA-B27 27% VKH 12.6% Behçet 2.5%	25%
Europe								
Italy [1] (2013–2015) [2017]	990	53.5% HSV 38.8% FUS 23.9% HLA-B27 19.1%	7.5% Pars planitis 89.2% Behçet 5.4% TB and sarcoidosis 2.7% each	16.2% PICCP 34.8% OT 27.8% TB 16.5%	22.8% Behçet 24.3% TB 23% Sarcoidosis 23%	30% of the 77% with an established diagnosis HSV 15.6% TB 5.7% OT 4.7%	70% of the 77% with an established diagnosis FUS 9.7% HLA-B27 7.7% Behçet 4.8%	33%
France [8] (1991–1996) [2001]	927	28.5% HSV/HZV 31% HLA-B27 17.4% Idiopathic 13.6%	15% Idiopathic 75.5% Multiple sclerosis 10.8% Sarcoidosis and Lyme disease 2.9%, each	21.6% OT 39% Birdshot 20.5% Idiopathic 16.5%	35% Idiopathic 37.7% Behcet 13.9% Sarcoidosis 10.5%	31.8%	34.2%	34%
England [28] (1991–2013) [2015]	3000	46% FUS 24.8%	11.1% Idiopathic 70.5%	21.8% Idiopathic 42.8%	21.1% Idiopathic 42.1%	NR OT 6.9%	NR FUS 11.5% Sarcoidosis 9.7%	-
Germany [29] (2001–2006) [2009]	1916	45.4% HLA-B27 15.4% FUS 11.3% Ankylosing spondylitis 9.5%	22.9% Idiopathic 53.7% MS 10.3% Sarcoidosis 7.8% FUS 7.3%	13.5% OT 24.7% Herpes 5.8% APMPPE 5%	6.2% Behçet 12.6% OT 11.77% Sarcoidosis 10.9%	22% Herpes 42.9 OT 34.6%	Systemic disease: 41.8% Ocular Syndrome:36.9%	35.3%
Africa								
Tunisia [32] (1992–2003) [2005]	472	35.2% Idiopathic 35.5% Herpes 33.7% FUS 8.4% Ankylosing spondylitis 4.8%	15.5% Idiopathic 86.3% Sarcoidosis 8.2% MS 4.1%	28.2% OT 38.3% Behçet 12.8% Idiopathic 10.5% Serpiginous choroiditis 5.3%	21.2% Behçet 36% Idiopathic 30% VKH 15% Multifocal choroiditis and panuveitis 8%	29% Herpes 11.9% OT 10.1% Toxocariasis 1.5%	35.1% Systemic disease 20.1% Behçet 12.3% Ankylosing spondylitis 1.7% Sarcoidosis 1.7% Specific ocular condition 15% VKH 4.4% FUS 3% Multifocal choroiditis and panuveitis 1.7%	35.2%
Oceania								
Australia [7] (2014–2015) [2019]	1236	74.4% HLA-B27 38.7% Herpes simplex 20.8% Ankylosing Spondylitis 13%	5.8% Sarcoidosis 45.4% TB 13.6% MS 9.0% HLA-B27 9.0%	15.2% OT 27.5% BD 13.2% VKH 12.2%	4.5% Sarcoidosis 44% VKH 24% Behçet 12%	13.4% Herpes simplex 44.2% Herpes zoster 19.3% OT 17.5%	Systemic disease 26.46% HLA-B27 42.5% Ankylosing spondylitis 13.7% Sarcoidosis 13.4%	60.19%

*NR*, not reported; *CMV*, cytomegalovirus; *DM*, diabetes mellitus; *FUS*, Fuchs’ uveitis syndrome; *HSV*, herpes simplex virus; *MEWDS*, multiple evanescent white dot syndrome; *OT*, ocular toxoplasmosis; *PICCP*, primary inflammatory choriocapillaropathies; *PSS*, Posner–Schlossman syndrome; *SO*, sympathetic ophthalmia; *SO*, sympathetic ophthalmia; *SSS*, seronegative spondyloarthropathies spectrum; *TB*, tuberculosis; *VKH*, Vogt-Koyanagi-Harada syndrome

## Conclusion

This study shows changes in the distribution pattern of uveitis, which can be explained by multiple reasons, such as the inclusion of new diagnostic technologies, socioeconomic changes, and the setting where the study is performed (specialized in retina vs. uveitis), among others. In Colombia, it is possible to ascertain that there has been a significant shift in the predominant causes of uveitis in all age groups in the last decades, from infectious diseases to immune-mediated etiologies. Two key factors could explain this; the first is that the patients examined in our current study had access to more diagnostic tools to detect a higher number of specific diagnoses than our Colombian cohort analyzed 15 years ago. The second is because the current study’s patients attended an ophthalmological center dedicated exclusively to uveitis, and in the previous one, patients attended uveitis and retina specialized centers. Therefore, it is recommended that all countries update their referring pattern studies to better understand the disease’s distribution by sex, age, and etiology. Thus, they can guide diagnosis, classification, and treatment more accurately and consequently avoid adverse outcomes such as blindness.

## Data Availability

The datasets used and/or analyzed during the current study are available from the corresponding author upon reasonable request.

## Abbreviations

BCVA: Best-corrected visual acuity
DANE: National Administrative Department of Statistics
HIV: Human immunodeficiency virus
HLA-B27: Human leukocyte antigen B27
OT: Ocular toxoplasmosis
SUN: Standardization of uveitis nomenclature
STROBE: Strengthening the reporting of observational studies in epidemiology
USA: United States of America
